# Interferon-γ-induced tolerogenic dendritic cells ameliorate autoimmune neuroinflammation and suppress T-cell responses in multiple sclerosis

**DOI:** 10.3389/fimmu.2026.1893121

**Published:** 2026-08-05

**Authors:** Constanza Vilchez, Brian Parra-Tello, Luis F. González, Viviana Noriega, Flavio Carrión, Rodrigo Pacheco, Carolina Prado, Eva Martínez-Cáceres, Carlos Guevara, Rodrigo Naves

**Affiliations:** 1 Interdisciplinary Nucleus of Pharmacology and Immunology, Instituto de Ciencias Biomédicas (ICBM), Facultad de Medicina, Universidad de Chile, Santiago, Chile; 2 Oficina de Apoyo a la Investigación Clínica (OAIC), Facultad de Medicina, Hospital Clínico Universidad de Chile, Santiago, Chile; 3 Department of Research and Innovation, Faculty of Health Sciences, Universidad del Alba, Santiago, Chile; 4 Facultad de Medicina, Universidad San Sebastián, Santiago, Chile; 5 Laboratorio de Neuroinmunología, Centro Científico y Tecnológico de Excelencia Ciencia & Vida, Fundación Ciencia & Vida, Santiago, Chile; 6 Department of Cell Biology, Physiology and Immunology, Universitat Autònoma de Barcelona (UAB), Cerdanyola del Vallés, Barcelona, Spain; 7 Immunology Department, Laboratori Clínic de la Metropolitana Nord (LCMN), Germans Trias I Pujol University Hospital, Badalona, Catalonia, Spain; 8 Department of Neurology and Neurosurgery, Hospital Clínico Universidad de Chile, Santiago, Chile

**Keywords:** experimental autoimmune encephalomyelitis (EAE), immune tolerance, interferon-gamma, multiple sclerosis, neuroinflammation, tolerogenic dendritic cells

## Abstract

**Background:**

Multiple sclerosis (MS) is a chronic autoimmune disease of the central nervous system characterized by immune-mediated demyelination and neuroinflammation resulting from the breakdown of self-tolerance. Tolerogenic dendritic cells (tolDC) have emerged as a promising therapeutic strategy to restore antigen-specific immune tolerance; however, generating stable tolDC under inflammatory conditions remains challenging. Although interferon (IFN)-γ is traditionally regarded as a pro-inflammatory cytokine, accumulating evidence indicates that it can also exert protective and immunoregulatory functions in specific cellular and disease contexts. Here, we investigated whether low-dose IFN-γ directly programs dendritic cells (DC) toward a stable tolerogenic phenotype with therapeutic relevance for MS.

**Methods:**

Murine bone marrow-derived DC were differentiated in the presence of IFN-γ and evaluated for phenotype, cytokine production, and T-cell modulatory capacity. Therapeutic efficacy was assessed in experimental autoimmune encephalomyelitis (EAE). Translational relevance was examined using monocyte-derived DC from treatment-naïve MS patients and healthy donors (HD).

**Results:**

IFN-γ-conditioned DC displayed reduced expression of co-stimulatory molecules and MHC-II, increased PD-L1 expression, and diminished pro-inflammatory cytokine production. Functionally, these cells suppressed myelin antigen-driven CD4^+^ T-cell proliferation and activation while promoting FoxP3^+^ regulatory T-cell differentiation. Adoptive transfer of myelin-derived peptide-loaded IFN-γ-conditioned DC significantly ameliorated established EAE and induced sustained clinical improvement, even after inflammatory challenge, demonstrating phenotypic and functional stability. In human studies, IFN-γ induced comparable tolerogenic phenotypes in DC from MS patients and HD; however, only MS-derived IFN-γ-conditioned DC effectively suppressed CD4^+^ and CD8^+^ T-cell responses. Residual CD80 expression correlated with T1 lesion burden.

**Conclusion:**

Collectively, these findings uncover a previously underappreciated context-dependent immunoregulatory role of IFN-γ in DC biology and immune tolerance. Furthermore, IFN-γ-induced DC emerge as a promising therapeutic strategy to restore immune tolerance in MS.

## 1 Introduction

Multiple sclerosis (MS) is a chronic, autoimmune, and neurodegenerative disorder of the central nervous system (CNS) characterized by immune-mediated demyelination, axonal injury, and progressive neuroinflammation (1, 2). Approximately 2.8 million individuals worldwide are affected, making MS one of the leading causes of neurological disability in young adults (3). The disease typically manifests between 20 and 40 years of age and occurs approximately twice as frequently in women as in men (4). Clinically, MS is classified into relapsing-remitting (RRMS), secondary progressive (SPMS), and primary progressive (PPMS) forms, which differ in their temporal patterns and progression of neurological impairment (5–7). Although its precise etiology remains incompletely understood, current evidence indicates that the disease results from a complex interplay between genetic susceptibility, environmental triggers, and dysregulated immune response that ultimately leads to the breakdown of self-tolerance and CNS-directed autoimmunity (8, 9). Experimental autoimmune encephalomyelitis (EAE) represents the most widely used animal model for investigating MS pathogenesis and evaluating candidate therapeutic strategies. EAE is a T-cell-mediated autoimmune disease induced by immunization with myelin-derived antigens such as myelin oligodendrocyte glycoprotein (MOG) peptide, proteolipid protein, or myelin basic protein. Disease development involves a peripheral inductive phase characterized by activation of autoreactive lymphocytes, followed by an effector phase marked by infiltration of inflammatory immune cells into the CNS and culminating in a chronic stage associated with persistent neuroinflammation, demyelination, and axonal damage (10–12).

Dendritic cells (DC) are professional antigen-presenting cells (APC) that orchestrate the balance between immune activation and immune tolerance. Depending on environmental cues, DC can acquire either an immunogenic or a tolerogenic phenotype. Tolerogenic DC (tolDC) are characterized by reduced expression of co-stimulatory molecules such as CD80, CD83, and CD86, decreased production of pro-inflammatory cytokines, and increased expression of inhibitory receptors that suppress effector T-cell activation while promoting regulatory T-cell (Treg) differentiation (13, 14). The adoptive transfer of tolDC has successfully restored immune tolerance in multiple models of autoimmunity, highlighting their translational potential as a cell-based therapy (15–17). Agents such as vitamin D_3_, inhibitors of JAK3/STAT5 signaling, dimethyl fumarate, atorvastatin, and synthetic triterpenoids have been shown to induce tolerogenic phenotypes in DC, both in murine models and in peripheral blood mononuclear cells from MS patients (18–27).

Interferon (IFN)-γ is a pleiotropic cytokine traditionally associated with proinflammatory immune response and the pathogenesis of autoimmune diseases, including MS. Nevertheless, accumulating evidence indicates that IFN-γ also exerts protective and immunoregulatory functions in specific cellular and disease contexts (28–31). We have previously demonstrated that administration of IFN-γ at the peak of EAE ameliorates disease symptoms and mitigates neuroinflammation through a STAT1-dependent mechanism. Histological analyses revealed reduced immune infiltration and demyelination in the spinal cords of IFN-γ–treated mice, accompanied by the induction of homeostatic and anti-inflammatory microglia (28). More recently, we demonstrated that IFN-γ induces a subset of splenic CD11b^+^ myeloid cells with tolerogenic properties capable of promoting Treg differentiation and limiting CNS infiltration of inflammatory T cells (32). Additional studies further suggest that IFN-γ may exert protective effects in EAE by modulating APC activity, inducing inhibitory molecules such as programmed death-ligand 1 (PD-L1) as well as regulatory cytokines, including IL-27, which suppress pathogenic T-cell responses and promote immune homeostasis (32, 33).

Based on these findings, we hypothesized that IFN-γ may directly program DC toward a tolerogenic phenotype, with therapeutic activity in EAE and immunomodulatory potential in MS. In the present study, we investigated this hypothesis by characterizing the phenotype, function, and therapeutic efficacy of IFN-γ–conditioned DC (IFN-γ-DC) in EAE and monocyte-derived DC (moDC) from MS patients and healthy donors (HD), aiming to elucidate their contribution to immune regulation and to explore their potential as a cytokine-based cell therapy for MS.

## 2 Methods

### 2.1 Animals

C57BL/6 mice were acquired from The Jackson Laboratory (Bar Harbor, ME) and housed at the specific pathogen-free Central Animal Facility of the Universidad de Chile. All experimental procedures were conducted in accordance with institutional guidelines for the care and use of laboratory animals and were approved by the Animal Care and Use Committee of the Faculty of Medicine, Universidad de Chile. 2D2 transgenic mice (expressing a TCR specific for the MOG_35–55_ peptide presented by I-A^b^) were purchased from The Jackson Laboratory (Bar Harbor, ME). OT-II transgenic mice (expressing TCRs specific for OVA_323–339_ presented by I-A^b^) were kindly donated by Dr. María Rosa Bono. Mice were euthanized by inhalation of isoflurane in a closed induction chamber containing a cotton pad soaked with 0.6 mL of liquid isoflurane, positioned to prevent direct contact between the animals and the anesthetic. Deep anesthesia was confirmed by the absence of the pedal withdrawal reflex following paw pinch with forceps, after which death was confirmed by cervical dislocation, in accordance with institutional animal care and use guidelines.

### 2.2 Generation of mouse bone marrow-derived dendritic cells

Bone marrow cells were collected from the femurs and tibias of 4–7 weeks old male or female C57BL/6 mice and suspended in complete RPMI 1640 medium (Gibco, California, US), supplemented with 1% penicillin-streptomycin (Gibco, California, US). After filtration through a 40 µm cell strainer, red blood cells were lysed with ammonium-chloride-potassium (ACK) buffer containing 0.15M NH_4_Cl, 10mM KHCO_3_, and 0.1mM Na_2_EDTA. Bone marrow precursors were cultured in 24-wells plates (1*10^6^ cell/well) in complete medium containing 20 ng/mL recombinant mouse granulocyte/macrophage-colony stimulating factor (GM-CSF; Biolegend, California, US) at 37 °C and 5% CO_2_. Half of the medium was replaced with fresh GM-CSF-containing medium on days 2, 4 and 6. Recombinant IFN-γ (5–50 ng/mL) was added at different stages of differentiation (day 2, 4 or 6) to generate IFN-γ-conditioned DC (IFN-γ-DC). DC differentiated without IFN-γ were used as immature DC (iDC) or they were stimulated with 100 ng/mL lipopolysaccharide (LPS; Sigma-Aldrich, Missouri, US) starting from day 6 to obtain mature DC (mDC). IFN-γ-DC were exposed to 100 ng/mL LPS during the last 24h (LPS-IFN-γ-DC) to determine phenotypic and functional stability.

### 2.3 Cytokine analysis by multiplex assays

Supernatants from BMDC cultures were analyzed for IL-1β, IL-12p70, IL-23, IL-17A, IL-6, TNF-α, IL-10, and IL-27 using a multiplex bead-based immunoassay (Millipore-Sigma, Saint Louis, US) according to the manufacturer’s instructions. Data were acquired with a Luminex^®^ 200™ System (Northbrook, Illinois, US).

### 2.4 T cell proliferation and activation assay

CD4^+^ T cells were isolated from the spleens of OT-II or 2D2 mice by negative selection using the CD4^+^ T cell Isolation Kit II (Miltenyi Biotec, North Rhine-Westphalia, Germany) according to the manufacturer’s instructions. Purified T cells were labeled with 2 µM of CellTrace™ Violet (CTV, Invitrogen, Massachusetts, USA) and co-cultured with mDC, IFN-γ-DC, or LPS-IFN-γ-DC at different DC: T cell ratios (2:1, 1:1, 1:2, 1:4) in 96-well U-bottom plates. Cells were stimulated with 20 ng/mL of OVA_323–339_ peptide (Genscript, New Jersey,USA) or 5 µg/mL of MOG_35–55_ peptide (CPC Scientific, California, USA) for 5 days at 37 °C and 5% CO_2_. CD4^+^ T cell proliferation and activation were determined by flow cytometry, measured as the percentage of divided cells and the frequency of CD4^+^CD25^+^ cells, respectively.

### 2.5 Treg cell induction

Total splenic CD4^+^ T cells from 2D2 mice were isolated by negative selection using the CD4^+^ T cell Isolation Kit II (Miltenyi Biotec, North Rhine-Westphalia, Germany) following the manufacturer’s instructions and co-cultured with mDC, IFN-γ-DC, or LPS-IFN-γ-DC previously loaded with 5µg/mL MOG_35–55_ peptide at a DC:T cell ratio of 1:4 for 5 days. Treg cell induction was determined by flow cytometry, evaluating the frequency of CD4^+^CD25^high^FoxP3^+^ cells.

### 2.6 EAE induction

EAE was induced in 9–12-week-old C57BL/6 mice by subcutaneous immunization with 150 μg MOG_35–55_ peptide (CPC Scientific, California, USA) emulsified in incomplete Freund’s adjuvant (IFA, BD Difco, Detroit, Michigan, USA) containing 500 μg Mycobacterium tuberculosis (BD Difco, Detroit, Michigan, USA). Mice received 500 ng *Bordetella pertussis toxin* (Cayman chemical company, Michigan, USA) intraperitoneally on days 0 and 2 post-immunization. Disease progression was monitored daily and scored as follows: 0, no symptoms; 1, limp tip of the tail; 2, complete tail atony; 3, partial paralysis of rear limbs; 4, total paralysis of hind legs; 5, severe paralysis, no movement (euthanasia is needed); 6, mouse is found dead.

### 2.7 Adoptive transfer of tolerogenic BMDC

IFN-γ-DC and LPS-IFN-γ-DC (1*10^6^ cells/dose) were pulsed with 5 µM of MOG_35–55_ peptide for 18 h and intravenously injected into EAE mice on days 14, 17, and 21 post-immunization. Control mice received PBS (vehicle).

### 2.8 Patients

Eleven treatment-naïve patients with MS, diagnosed according to the revised McDonald criteria (34) and the Lublin and Reingold criteria (5) were recruited from the Department of Neurology at the Hospital Clínico, Facultad de Medicina, Universidad de Chile. All patients underwent clinical examination and magnetic resonance imaging of the brain and spinal cord prior to inclusion. Disability was assessed using the Expanded Disability Status Scale (EDSS) (35). Patients with systemic inflammation, other inflammatory neurological diseases, or immunomodulatory therapy within 3 months before sampling were excluded. The study was approved by the Ethics Committee of the Hospital Clínico and Facultad de Medicina, Universidad de Chile. Written informed consent was obtained from all participants in accordance with the Declaration of Helsinki. Peripheral blood (80 mL) was obtained from each MS patient and HD into lithium heparin tubes (BD Biosciences, San Jose, CA).

### 2.9 Generation of human monocyte-derived dendritic cells

Peripheral blood mononuclear cells (PBMC) were isolated from MS patients (n=11) and HD (n=11) using Ficoll-Paque density gradient (Amersham, Uppsala, Sweden). Monocytes were purified using the Pan Monocyte Isolation Kit (Miltenyi Biotec, North Rhine-Westphalia, Germany), achieving > 90% purity. Cells were cultured in AIM-V medium (GIBCO, California, US) supplemented with recombinant human GM-CSF and IL-4 (1000 U/mL each; BioLegend, California, US) in 24-well plates (1×10^6^ cells/well) at 37 °C and 5% CO_2_. Half of the medium was replaced on days 2 and 4, with fresh cytokine-supplemented medium. Recombinant human IFN-γ (10 ng/ml) was added from day 0 to generate IFN-γ-moDC. moDC differentiated in the absence of IFN-γ were used as iDC. On day 5, 1µg/mL LPS (Sigma-Aldrich, Missouri, US) was added for 24 h to induce maturation (mDC).

### 2.10 Mixed lymphocyte reaction assay

PBMC from HD were labeled with 5µM CTV and co-cultured with mDC or IFN-γ-moDC derived from MS patients or HD at a 1:4 DC:T cell ratio. Cultures were stimulated with 2 µg/mL phytohemagglutinin (PHA, Roche, Basilea, Suiza) for 5 days at 37 °C and 5% CO_2_.

### 2.11 Flow cytometry

Cells were stained with Zombie UV fixable viability dye (Biolegend) for 15 min at room temperature. Fc receptors were blocked with anti-mouse CD16/32 antibody (TruStain FcX, Biolegend) for 10 min at 4 °C. Surface staining was performed using fluorochrome-conjugated antibodies listed in **Supplementary Table 1** for 30 min at 4 °C. For intracellular staining, cells were permeabilized with the FoxP3/Transcription Factor Staining Buffer Set (Invitrogen, Massachusetts, USA) for 45 min at 4 °C, followed by staining with intracellular antibodies for 25 min at 4 °C. Samples were acquired on an LSR FORTESSA flow cytometer (BD Bioscience, USA) and analyzed using FlowJo software (Tree Star, USA).

### 2.12 Statistical analysis

First, data were tested for normality to determine whether they followed a Gaussian distribution. Groups were subsequently compared using paired t-tests or one-way ANOVA for parametric data, and Wilcoxon or Friedman tests for nonparametric data. EAE clinical scores were analyzed using two-way ANOVA. Statistical analyses were performed using GraphPad Prism version 6.0 (GraphPad Software Inc., USA). Data are presented as mean ± SEM. A *P* value < 0.05 was considered statistically significant.

## 3 Results

### 3.1 IFN-γ treatment induces a stable tolerogenic phenotype in murine BMDC

**Figure 1:**
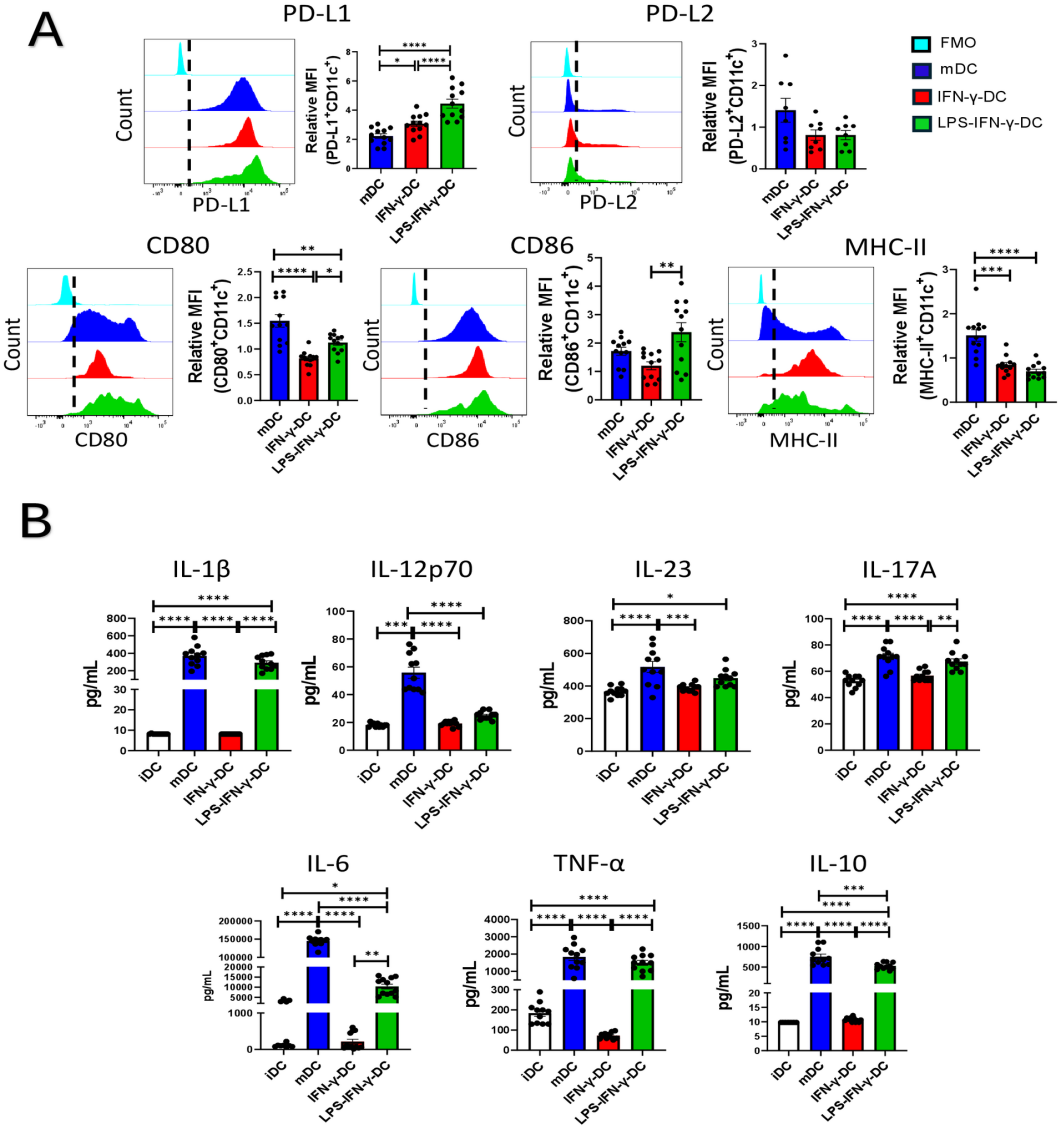
IFN-γ induces a stable tolerogenic phenotype in murine bone-marrow-derived dendritic cells. BMDCs were differentiated in the presence or absence of IFN-γ, added on day 2, and analyzed on day 7. **(A)** Surface expression of PD-L1, PD-L2, CD80, CD86, and MHC-II molecules was determined in LPS-matured DC (mDC, blue bars), IFN-γ-treated DC (IFN-γ-DC, red bars), and IFN-γ-DC exposed to LPS during the last 24 h of culture (LPS-IFN-γ-DC, green bars) (n=12). Representative flow cytometry histograms are shown. FMO, fluorescence minus one. Data were normalized to immature DC. **(B)** Secretion of IL-1β, IL-23, IL-17A, IL-10, IL-12p70, IL-6, TNF-α, and IL-27 was determined in cell culture supernatants by multiplex cytokine assay (n=11). Data are shown as mean ± SEM from three independent experiments. Statistical analysis was performed using one-way ANOVA with Bonferroni's post-hoc test. *P < 0.05; **P < 0.01; ***P < 0.001; ****P < 0.0001.

We first conducted a pilot study to determine the optimal conditions for mouse bone marrow-derived dendritic cells (BMDC) differentiation in response to interferon (IFN)-γ. BMDC were cultured in the presence of increasing concentrations of IFN-γ (5, 10, or 50 ng/ml) added on days 0, 2, or 4 of differentiation. Based on cell viability, differentiation yield, and the expression profiles of co-stimulatory and co-inhibitory molecules, we determined that 50 ng/mL IFN-γ added from day 2 was the optimal condition for BMDC differentiation (**Supplementary Figure 1**). To characterize the phenotype of IFN-γ–conditioned dendritic cells (IFN-γ-DC), we analyzed surface markers by flow cytometry. IFN-γ-DC displayed significantly higher expression of programmed death-ligand 1 (PD-L1) and reduced expression of CD80 and major histocompatibility complex (MHC)-II molecules compared to mature DC (mDC). PD-L2 and CD86 expression remained unchanged. Remarkably, this phenotype was maintained even after stimulation with lipopolysaccharide (LPS; LPS-IFN-γ-DC) compared to mDC (**Figure 1A**). Consistent with this phenotype, IFN-γ-DC secreted markedly lower levels of inflammatory cytokines, including IL-1β, IL-12p70, IL-23, IL-17A, IL-6, and TNF-α relative to mDC. In contrast, IL-10 production in IFN-γ-DC remained comparable to that observed in immature DC (iDC), indicating that IFN-γ conditioning did not enhance IL-10 secretion during DC differentiation. Notably, following LPS-induced maturation, LPS-IFN-γ-DC exhibited significantly lower levels of IL-12p70, IL-6, and IL-10 than mDC, consistent with attenuation of cytokine production induced by LPS-induced maturation (**Figure 1B**). Secretion of IL-27 was undetectable in all groups (data not shown).

### 3.2 Tolerogenic and therapeutic activity of IFN-γ-DC

**Figure 2:**
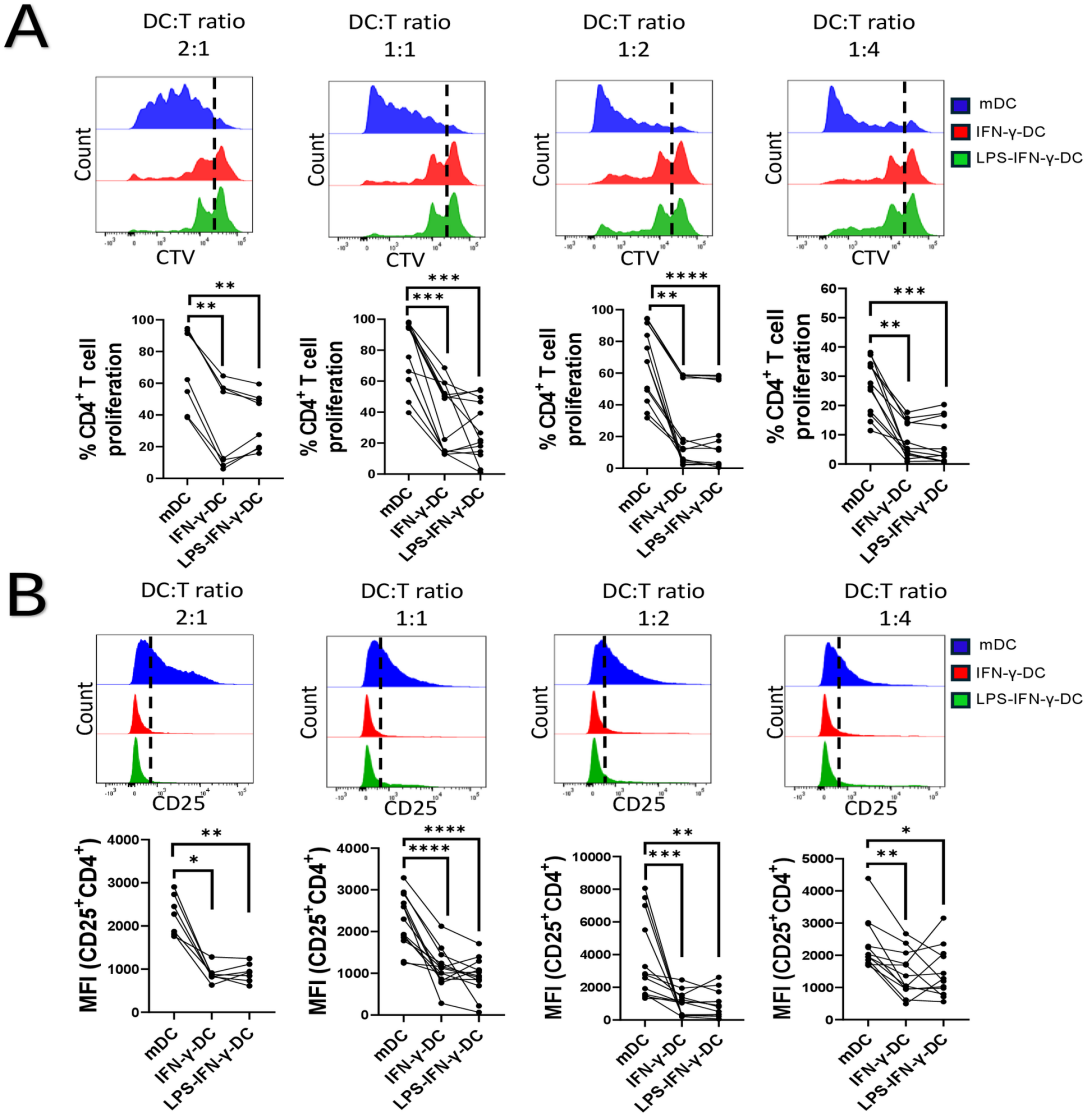
IFN-γ-DC suppress antigen-specific CD4^+^ T-cell proliferation and activation. CD4^+^ T cells isolated from OT-II mice were labeled with CellTrace Violet (CTV) and co-cultured with mature DC (mDC, blue bars), IFN-γ-treated DC (IFN-γ-DC, red bars), or IFN-γ-DC exposed to LPS during the last 24 h of culture (LPS-IFN-γ-DC, green bars) at different DC:T cell ratios in the presence of OVA_323–339_ peptide for 5 days. **(A)** CD4^+^ T cell proliferation was determined by CTV dilution by flow cytometry (n=7-13). **(B)** CD4^+^ T cell activation was evaluated by CD25 expression measured as mean fluorescence intensity (MFI) by flow cytometry (n=7-13). Data are shown as mean ± SEM from three independent experiments. Statistical analysis was performed using Friedman test with Wilcoxon signed-rank post-hoc test for non-parametric data or one-way ANOVA with Bonferroni's post-hoc test for parametric data. *P < 0.05; **P < 0.01; ***P < 0.001; ****P < 0.0001.

**Figure 3:**
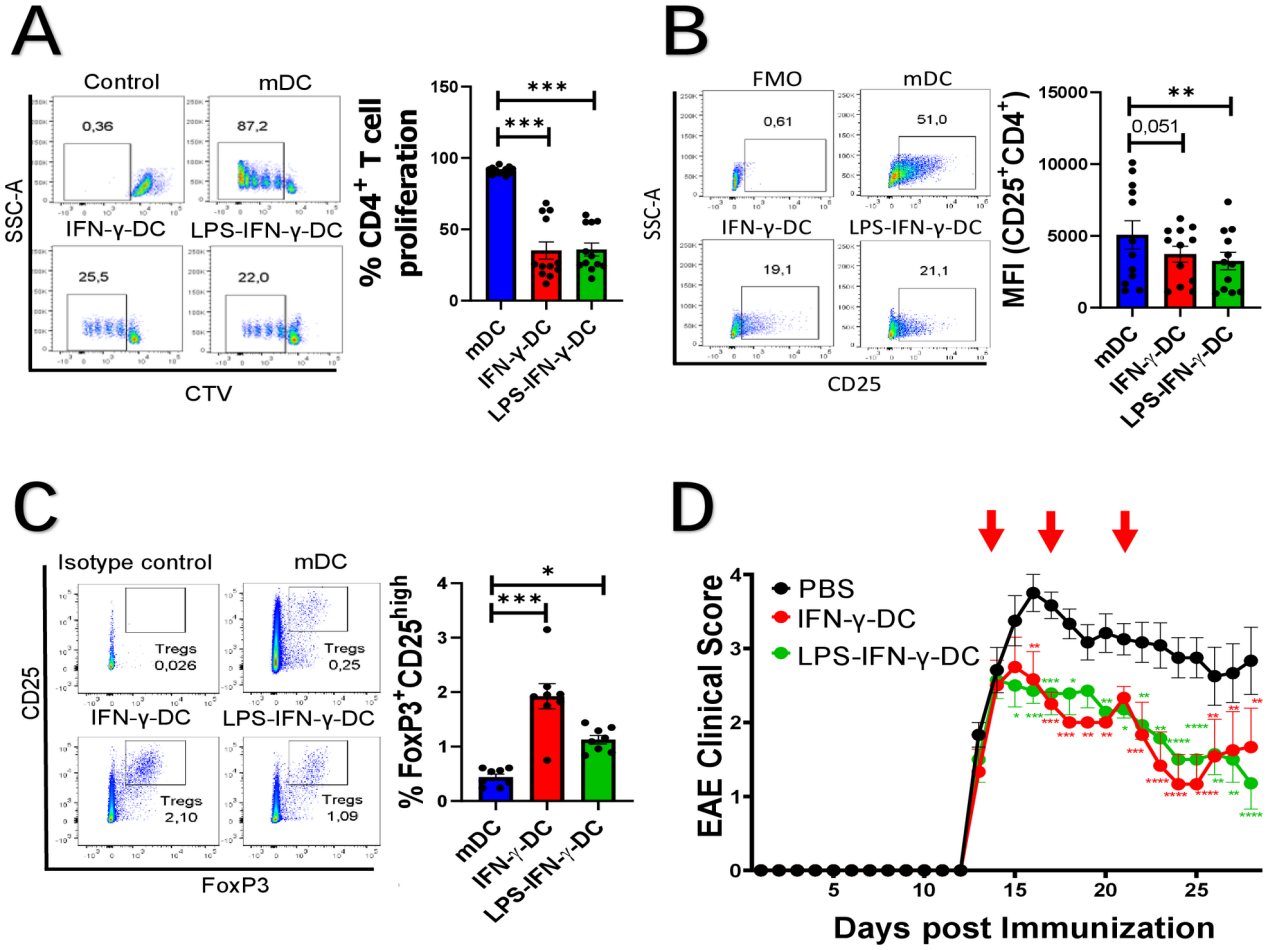
Tolerogenic and therapeutic activity of IFN-γ-DC. CD4^+^ T cells isolated from 2D2 mice were labeled with CellTrace Violet (CTV) and co-cultured with mature DC (mDC, blue bars), IFN-γ-treated DC (IFN-γ-DC, red bars), or IFN-γ-DC exposed to LPS during the last 24 h of culture (LPS-IFN-γ-DC, green bars) in the presence of MOG_35–55_ peptide at a DC:T cell ratio of 1:4 for 5 days. **(A)** CD4^+^ T cell proliferation was determined by CTV dilution by flow cytometry (n=12). **(B)** CD4^+^ T cell activation was measured by CD25 expression as mean fluorescence intensity (MFI) by flow cytometry (n=12). **(C)** Frequency of CD4^+^CD25^high^FoxP3^+^ Treg cells (n=8). Data are presented as mean ± SEM from three independent experiments. **(D)** PBS (vehicle control, black circles), IFN-γ-treated DC (IFN-γ-DC, red circles), or IFN-γ-DC subsequently stimulated with LPS during the last 24 h of culture (LPS-IFN-γ-DC, green circles) (1×10^6^ cells/dose) were pulsed with 5 µM of MOG_35–55_ peptide for 18 h and administered intravenously to EAE mice on days 14, 17, and 21 post-immunization (red arrows). Clinical scores were recorded daily until day 28. Data are presented as mean ± SEM. n= 6–7 mice/group. For panels **A–C**, data are presented as mean ± SEM from three independent experiments. Statistical analysis was performed using the Friedman test, followed by the Wilcoxon signed-rank post-hoc test for nonparametric data or the one-way ANOVA, followed by Bonferroni's post-hoc test for parametric data. For panel D, statistical analysis was performed using two-way ANOVA followed by Bonferroni’s *post-hoc* test. *P < 0.05; **P < 0.01; ***P < 0.001; ****P < 0.0001.

To assess the functional activity of IFN-γ-DC, we determined whether these cells could induce antigen-driven immune tolerance. Splenic CD4^+^ T cells from OT-II mice were labeled with 2 µM CTV, and co-cultured with mDC, IFN-γ-DC, or LPS-IFN-γ-DC at different DC:T cell ratios in the presence of OVA_323–339_ peptide. The results showed that both IFN-γ-DC and LPS-IFN-γ-DC significantly suppressed CD4^+^ T cell proliferation (**Figure 2A**) and reduced T cell activation (**Figure 2B**) at all tested DC:T cell ratios. We next evaluated the tolerogenic capability of myelin oligodendrocyte glycoprotein (MOG)_35–55_-loaded IFN-γ-DC. Consistent with the above results, both IFN-γ-DC and LPS-IFN-γ-DC significantly inhibited CD4^+^ T cell proliferation (**Figure 3A**). LPS-IFN-γ-DC significantly inhibited CD4^+^ T cell activation, whereas IFN-γ-DC exhibited a comparable inhibitory trend that did not reach statistical significance (*P* = 0.051) (**Figure 3B**). Remarkably, both IFN-γ-DC and LPS-IFN-γ-DC promoted a significant conversion of CD4^+^ T cells into regulatory T (Treg) cells compared to mDC (**Figure 3C**). To determine the *in vivo* therapeutic potential of IFN-γ-DC, 1×10^6^ MOG-loaded IFN-γ-DC or LPS-IFN-γ-DC were adoptively transferred into experimental autoimmune encephalomyelitis (EAE) mice on days 14, 17, and 21 post-immunization, when animals had already developed established clinical signs of disease. Both IFN-γ-DC and LPS-IFN-γ-DC induced a significant reduction in EAE clinical scores compared with PBS-treated mice. This effect became evident shortly after the first administration and persisted throughout the study period, including seven days after the final dose (**Figure 3D**).

### 3.3 IFN-γ induces a tolerogenic phenotype in human moDC from MS patients and HD

**Figure 4:**
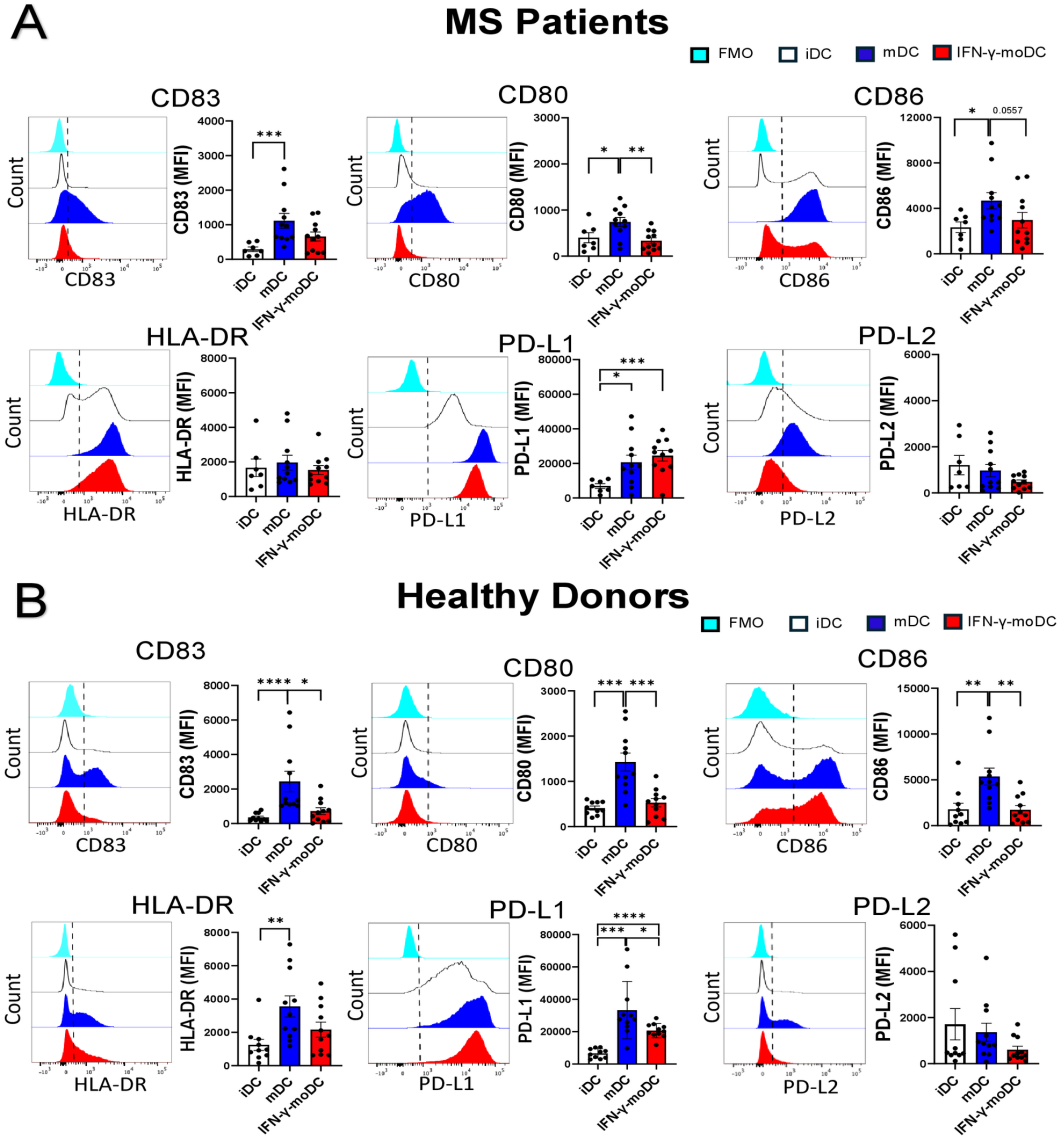
IFN-γ induces a tolerogenic phenotype in human monocyte-derived DC from MS patients and HD. Peripheral blood monocytes from treatment-naïve MS patients (n=7-11) or healthy donors (HD, n=10-11) were differentiated into iDC (white bars), mDC (blue bars), or IFN-γ-moDC (red bars). Surface expression of CD83, CD80, CD86, HLA-DR, PD-L1, and PD-L2 was analyzed by flow cytometry in **(A)** MS-derived moDC and **(B)** HD-derived moDC. Data are shown as mean ± SEM. Statistical analysis was performed using paired t-test for parametric data or Wilcoxon test for non-parametric data. *P < 0.05; **P < 0.01; ***P < 0.001; ****P < 0.0001.

To extend our findings to humans, we performed an exploratory analysis to assess the impact of IFN-γ on the differentiation of monocyte-derived DC (moDC) generated from PBMC of multiple sclerosis (MS) patients and healthy donors (HD). The demographic and clinical characteristics of MS patients and HD are summarized in **Table 1**. As expected, mDC from MS patients showed significantly higher CD83 levels than iDC. In contrast, IFN-γ-moDC exhibited intermediate levels of CD83 compared with iDC and mDC, consistent with a semi-mature phenotype. IFN-γ-moDC exhibited CD80 levels significantly lower than those observed in mDC and comparable to those of iDC. Similarly, CD86 expression tended to be lower than in mDC, although this difference did not reach statistical significance (*P* = 0.0557). PD-L1 expression was comparable to that observed in mDC and significantly higher than in iDC, whereas HLA-DR and PD-L2 remained unchanged (**Figure 4A**). A similar profile was observed in HD-derived moDC. IFN-γ-moDC exhibited a phenotype consistent with tolerogenic DC, characterized by levels of CD83, CD80, and CD86 that were significantly lower than those observed in mDC and comparable to those of iDC. In addition, PD-L1 expression was significantly higher than in iDC, although remaining lower than that observed in mDC. HLA-DR and PD-L2 expression did not differ between IFN-γ-moDC and the other conditions (**Figure 4B**).

**Table 1:** Demographic and clinical data of MS patients and HD.

Characteristics	Category	Patients	HD
Number		11	11
Gender	Male	1	4
	Female	10	7
Mean age (years)		31,4 ± 5,7	32,3 ± 2,8
Age at onset (years)		26,9 ± 4,9	n.a.
Disease type	RRMS	10	n.a.
	PPMS	1	n.a.
Mean EDSS		2,3 ± 1,7	n.a.
Average untreated time (months)		17,5 ± 19,1	n.a.
Previous treatment	Rebiff	4	n.a.
	Avonex	1	n.a.
	Methylprednisolone	1	n.a.
	Untreated	5	n.a.
T1 lesions		2,7 ± 2,9	n.a.
T2 lesions		16,2 ± 17,4	n.a.

n.a= not applicable.

### 3.4 IFN-γ-moDC differentially suppress CD4^+^ and CD8^+^ T cell proliferation and activation in MS patients and HD

**Figure 5:**
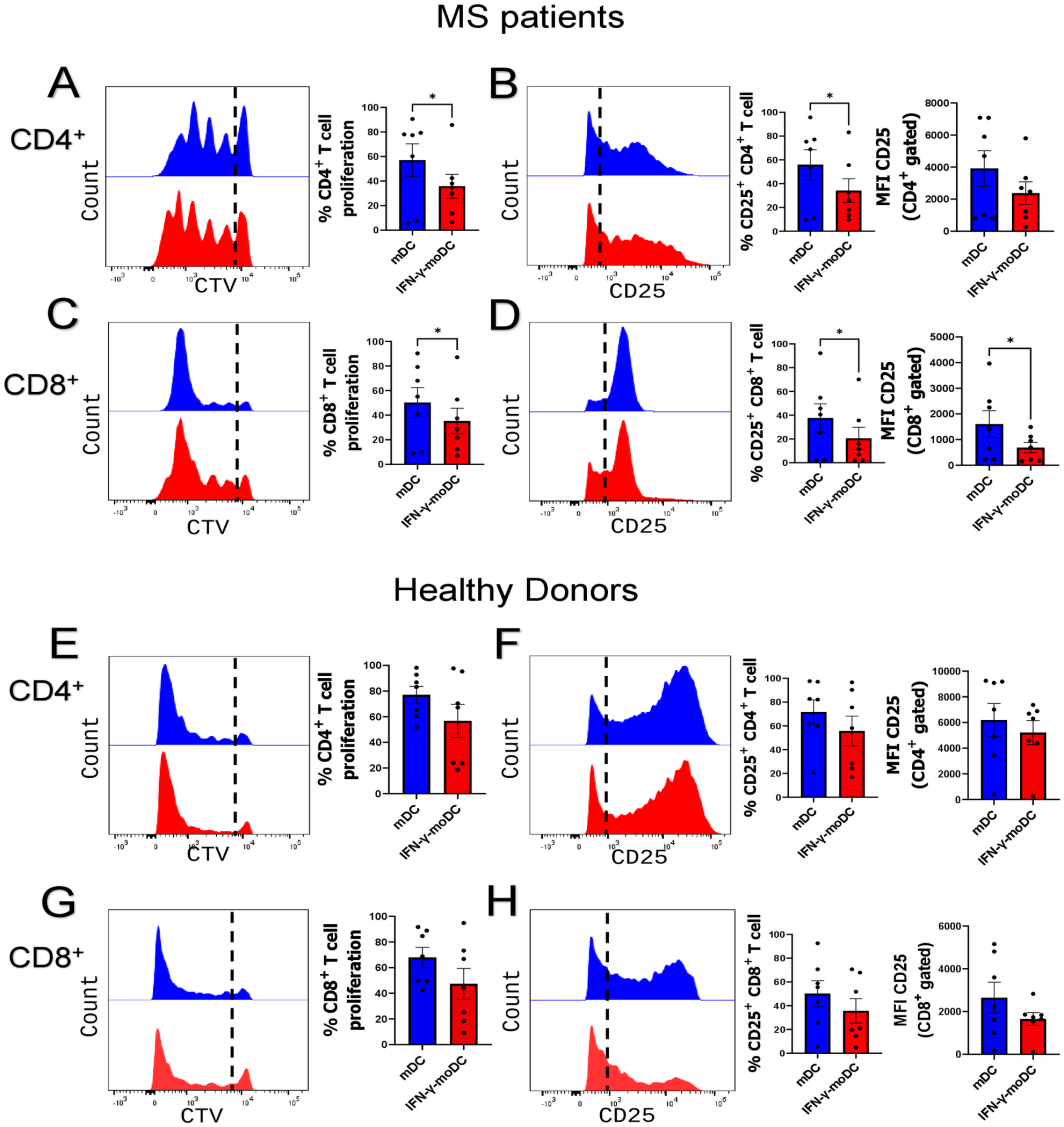
IFN-γ-moDC from MS patients suppress allogeneic CD4^+^ and CD8^+^ T-cell proliferation and activation. IFN-γ-moDCs or mDCs from treatment-naïve MS patients (n=7) or healthy donors (HD, n=7) were co-cultured with CellTrace Violet (CTV)-labeled allogeneic PBMC and stimulated with 2 µg/mL phytohemagglutinin for 5 days. **(A, C)** CD4^+^ and CD8^+^ T cell proliferation and **(B, D)** activation in MS-patient derived cultures were determined by flow cytometry. **(E, G)** CD4^+^ and CD8^+^ T cell proliferation and **(F, H)** activation in HD-derived cultures were determined by flow cytometry. Data are presented as mean ± SEM. Statistical analysis was performed using paired t-test for parametric data or Wilcoxon test for non-parametric data. *P < 0.05.

To evaluate the tolerogenic function of IFN-γ-moDC derived from MS patients and HD, a MLR assay was performed. IFN-γ-moDC generated from a subset of treatment-naïve MS patients and HD were co-cultured with allogenic PBMC and stimulated with PHA for 5 days. The results showed that, in MS patients, IFN-γ-moDC significantly inhibited the proliferation of both CD4^+^ and CD8^+^ T cells compared to mDC (**Figures 5A, C**). Consistently, IFN-γ-moDC also induced a significant reduction in the frequency of activated CD4^+^ and CD8^+^ T cells. In addition, IFN-γ-moDC induced significantly lower CD25 expression in CD8^+^ T cells compared with mDC. Although a reduction in CD25 expression in CD4^+^ T cells was also observed, this difference did not reach statistical significance (P = 0.0781) (**Figures 5B, D**). In contrast, IFN-γ-moDC derived from HD did not significantly affect the proliferation or activation of CD4^+^ and CD8^+^ T cells compared with mDC (**Figures 5E–H**). Analysis of cell death pathways revealed that IFN-γ conditioning did not significantly affect apoptosis or necrosis in CD4^+^ or CD8^+^ T cells from either MS patients or HD. The only exception was a significant reduction in apoptosis of CD4^+^ T cells induced by IFN-γ-moDC from MS patients (**Supplementary Figure 2**).

### 3.5 Association between clinical parameters and immune profile in MS patients

**Figure 6:**
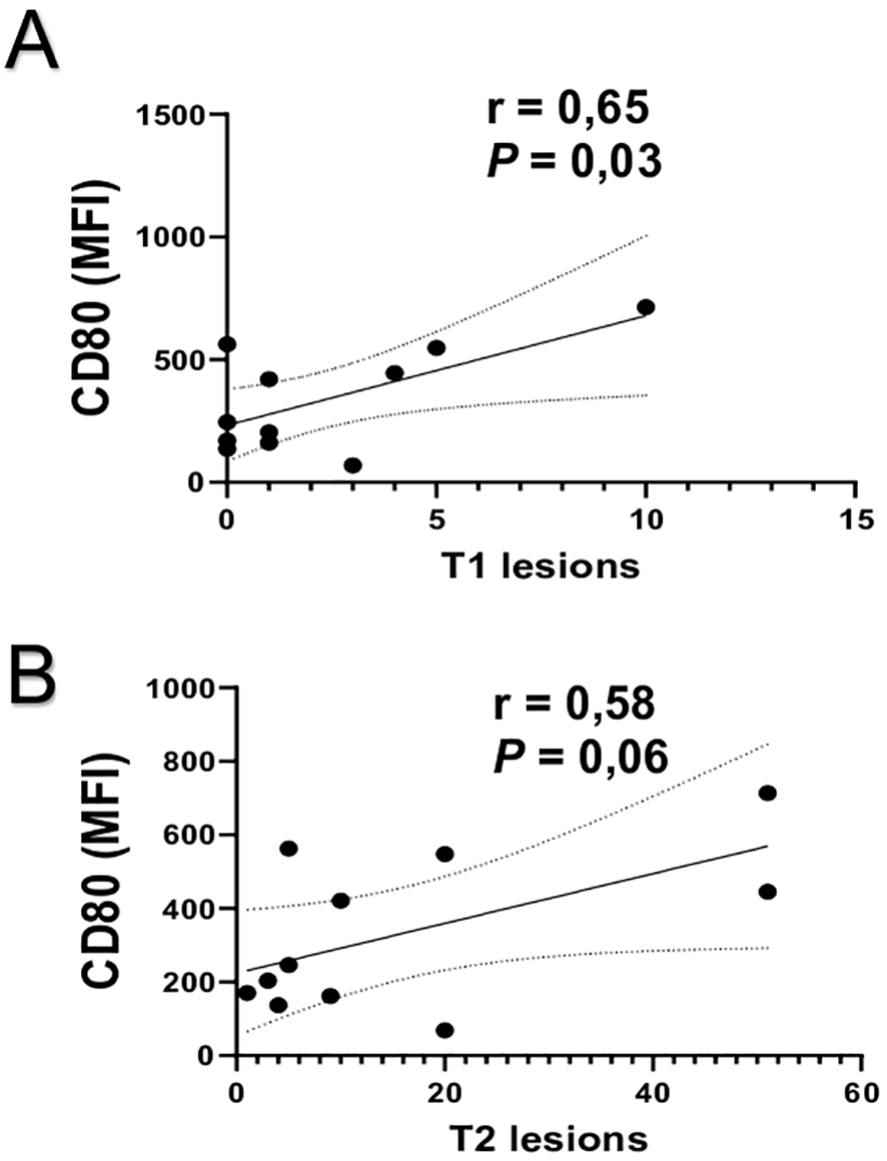
Association between MRI-derived lesion burden and DC immunophenotype in MS. Correlation analyses were performed to evaluate the association between MRI parameters and the immune phenotype of IFN-γ-moDC derived from MS patients (n=11). Associations between **(A)** CD80 expression and T1 lesion burden, and **(B)** CD80 expression and T2 lesion burden are shown. Pearson or Spearman correlation coefficients **(r)** and corresponding *P* values are indicated in each panel.

To explore the clinical relevance of the IFN-γ-induced immune phenotype in moDC from MS patients, we examined the association between DC immunophenotype and radiological/clinical parameters, including T1- and T2-weighted lesion burden and disability score (EDSS). In IFN-γ-moDC, CD80 expression showed a significant positive correlation with T1 lesion burden (**Figure 6A**). CD80 expression also showed a positive trend toward association with T2 lesion burden that did not reach statistical significance (*P* = 0.06) (**Figure 6B**). No significant correlations were observed between CD83 or PD-L1 expression and T1 lesion burden (**Supplementary Figure 3A**), nor between CD80, CD83, or PD-L1 expression and T2 lesion burden (**Supplementary Figure 4A**). Likewise, no significant associations were detected in mDC (**Supplementary Figures 3B**, **4B**). Finally, no significant associations were observed between DC immunophenotype and EDSS in either IFN-γ-moDC or mDC (**Supplementary Figure 5**).

## 4 Discussion

In this study, we demonstrate that IFN-γ, traditionally viewed as a pro-inflammatory cytokine, can act as a context-dependent immunoregulatory cytokine (28–32), directly reprogramming murine BMDC toward a tolerogenic phenotype with therapeutic efficacy in EAE. Murine IFN-γ-conditioned DC exhibited a phenotypic and functional profile characterized by lower expression of co-stimulatory molecules, elevated PD-L1 expression, broad modulation of cytokine production, and a robust capacity to inhibit effector T-cell responses while promoting regulatory T-cell differentiation. The ability of IFN-γ-DC to ameliorate established EAE underscores their therapeutic potential. Furthermore, the persistence of clinical benefit throughout the treatment period and for at least seven days after the final administration suggests that IFN-γ-driven tolerogenic reprogramming can effectively rebalance pathogenic and regulatory immune responses during active disease. Remarkably, the phenotype, functional profile, and therapeutic activity of IFN-γ-DC remained stable even after exposure to an inflammatory stimulus such as LPS, a crucial feature for the clinical translation of tolerogenic DC (25, 36–38). Extending these findings to humans, IFN-γ also promoted a tolerogenic profile in moDCs from both MS patients and HD, characterized by expression levels of maturation-associated co-stimulatory molecules that were lower than those observed in mDC and comparable to those of iDC, together with elevated PD-L1 expression relative to iDC, and the capacity to suppress CD4^+^ and CD8^+^ T-cell proliferation and activation in MS. Collectively, these phenotypic and functional features support the induction of a tolerogenic phenotype.

Mechanistically, murine IFN-γ-DC were characterized by broad modulation of cytokine production, including reduced secretion of key mediators involved in Th1 and Th17 differentiation, such as IL-12p70, IL-23, and IL-6. In addition, IFN-γ-DC exhibited increased PD-L1 expression relative to iDC. Notably, IFN-γ conditioning did not increase IL-10 production during DC differentiation, as IFN-γ-DC produced IL-10 levels comparable to those observed in iDC. Furthermore, LPS-IFN-γ-DC exhibited lower levels of IL-12p70, IL-6, and IL-10 than mDC, indicating that IFN-γ attenuates the increase in these cytokines associated with LPS-induced maturation. While the reduced production of IL-12p70 and IL-6 under inflammatory conditions is consistent with the acquisition of a tolerogenic phenotype, the concomitant reduction in IL-10 indicates that the suppressive activity of IFN-γ-DC cannot be attributed to enhanced secretion of this regulatory cytokine. Similarly, IL-27 was undetectable in all experimental groups. Together, these findings indicate that, in our experimental system, the tolerogenic phenotype induced by IFN-γ is not associated with increased production of regulatory cytokines such as IL-10 or IL-27. A previous study has shown that the IFN-γ/IL-27 axis induces PD-L1 expression in moDC and tolerance in CNS autoimmunity (33). However, the absence of detectable IL-27 in our system suggests that this pathway is unlikely to account for the increased PD-L1 expression and tolerogenic activity observed in IFN-γ-DC. Supporting an alternative mechanism, previous studies from our group demonstrated that genetic ablation of STAT1 abolished IFN-γ-driven tolerogenic reprogramming of both microglia and splenic myeloid cells, as well as the therapeutic effects of IFN-γ in EAE, identifying the IFN-γ/STAT1 axis as a central pathway mediating immune regulation and tolerance (28, 32). Consistent with this concept, several studies have shown that IFN-γ directly induces PD-L1 expression in antigen-presenting cells through STAT1-dependent transcriptional programs (39, 40). Together, these findings suggest that activation of the IFN-γ/STAT1 pathway may also contribute to the induction of a tolerogenic phenotype in DC. Future studies incorporating genetic, transcriptional, and pathway-specific approaches will be required to define the contribution of STAT1 and additional regulatory pathways, including IDO1 and SOCS family members, to the establishment and maintenance of DC tolerance.

Collectively, our findings suggest that IFN-γ promotes immune tolerance by establishing a tolerogenic DC program characterized by reduced antigen-presenting capacity, increased expression of regulatory surface molecules, and suppression of pathogenic T-cell responses. The MOG_35-55_-specific proliferation assay demonstrated that murine IFN-γ-DC suppress the proliferation and activation of MOG-reactive CD4^+^ T cells. The concomitant increase in FoxP3^+^ Treg cells further supports the notion that IFN-γ-DC promote the differentiation of T cells toward a regulatory phenotype. Importantly, LPS-treated IFN-γ-DC retained suppressive and Treg-inducing capacity, reinforcing the notion that IFN-γ confers a resilient tolerogenic program rather than a transient functional state.

Adoptive transfer of MOG-loaded IFN-γ-DC significantly ameliorated EAE clinical symptoms, with clinical improvement maintained throughout the observation period, including at least seven days after the final administration. These findings indicate that IFN-γ-conditioned DC can restore immune tolerance *in vivo* and are consistent with a previous study showing that IFN-γ-modulated splenic DC suppress EAE and induce apoptosis of myelin-reactive T cells (41). The persistence of clinical benefit during the study period suggests that IFN-γ-DC may promote durable immunoregulatory mechanisms capable of controlling ongoing autoimmune responses. Moreover, the therapeutic benefit was achieved despite administration at the peak of disease, demonstrating that IFN-γ-conditioned DC remain effective even after clinical disease has been established.

The encouraging therapeutic efficacy observed in the present study is supported by the growing clinical translation of tolerogenic DC-based therapies for MS. Initial phase I studies demonstrated the feasibility and safety of autologous myelin peptide-loaded vitamin D_3_-generated tolerogenic DC (42), and this therapeutic strategy has subsequently progressed through harmonized phase I studies (43) to phase II clinical evaluation (ClinicalTrials.gov Identifier: NCT07020715). These recent clinical advances further support the translational relevance of developing alternative strategies for generating therapeutically effective tolerogenic DC, such as IFN-γ-conditioned DC.

Remarkably, IFN-γ-conditioned moDCs from both MS patients and HD recapitulated key phenotypic features of the tolerogenic profile observed in murine systems, characterized by expression levels of maturation-associated co-stimulatory molecules that were lower than those observed in mDC and comparable to those of iDC along with elevated PD-L1 expression relative to iDC. Supporting our findings, Svajger et al. demonstrated IFN-γ-mediated induction of PD-L1 on human moDC from HD (44). However, despite these similar phenotypic changes, functional assays revealed an important divergence between MS patients and HD. IFN-γ-moDC derived from MS patients effectively inhibited CD4^+^ and CD8^+^ T cell proliferation and activation, whereas IFN-γ-moDC from HD failed to significantly modulate these responses. This differential functional response observed between MS patients and HD further supports the concept of context-dependent IFN-γ activity. This observation suggests that acquisition of a tolerogenic surface phenotype alone may not fully predict the immunoregulatory capacity of human moDC. Instead, disease-associated factors may influence the downstream functional consequences of IFN-γ signaling. In MS, chronic inflammatory priming may condition myeloid cells to respond to IFN-γ by activating regulatory pathways that enhance their tolerogenic potential. Under these conditions, the coordinated modulation of surface molecules and regulatory pathways induced by IFN-γ may more effectively promote inhibitory signals and limit T-cell activation, proliferation, and effector responses. Future studies incorporating cytokine profiling, transcriptional analyses, and additional functional characterization will be important to determine whether distinct molecular programs underlie the divergent responses observed between MS patients and HD. From a translational perspective, these findings suggest that IFN-γ-mediated cellular reprogramming may be particularly effective in restoring immune regulation in autoimmune settings such as MS.

A limitation of the human moDC experiments is that the stability of the IFN-γ-induced tolerogenic phenotype under inflammatory conditions could not be directly assessed, as the limited number of monocytes obtained from MS patients and HD precluded the inclusion of an additional LPS-challenged IFN-γ-moDC group.

Importantly, exploratory analyses identified clinically relevant association between the IFN-γ-conditioned moDC immune phenotype and MRI-derived measures of disease burden in MS patients. Residual CD80 expression in IFN-γ-moDC was positively correlated with T1 lesion burden and showed a trend toward a positive association with T2 lesion load. T1 lesions are commonly associated with axonal damage and irreversible tissue injury, whereas T2 lesions reflect cumulative inflammatory burden within the CNS (45, 46). These findings suggest that incomplete suppression of co-stimulatory signaling in IFN-γ-moDC may be linked to both chronic tissue damage and overall lesion accumulation.

However, these findings should be interpreted cautiously, as the observed associations do not establish causality and were derived from a relatively small cohort of treatment-naïve MS patients. Moreover, the correlation with T2 lesion burden did not reach statistical significance and should therefore be considered exploratory. Validation of these findings in larger, independent cohorts of treatment-naïve MS patients, including adequately powered analyses according to disease subtype and clinical activity, will be necessary to establish their generalizability and potential clinical significance.

The absence of significant associations between DC immune phenotype and disability scores (EDSS) is not unexpected, given the relatively low disability values observed in the analyzed cohort. In this context, MRI-derived lesion measures may provide a more sensitive readout of immune–CNS interactions at earlier stages of disease progression (47, 48).

Collectively, our results are consistent with accumulating evidence showing that IFN-γ can reprogram myeloid cells and microglia toward a regulatory profile, even in inflammatory or autoimmune contexts (28, 32, 44, 49). Our findings extend this concept and uncover a previously underappreciated regulatory role of IFN-γ in DC biology, demonstrating that IFN-γ not only reshapes DC phenotype but also modifies their functional interactions with T cells in a disease-dependent manner, favoring immune homeostasis in MS. The capacity of IFN-γ to restore tolerogenic competence in DC derived from MS patients underscores its therapeutic promise as a cytokine-based cellular reprogramming strategy. This approach could provide a personalized and physiologically relevant means of generating autologous tolDC for cell therapy in MS and potentially other autoimmune neuroinflammatory diseases.

## Data Availability

The raw data supporting the conclusions of this article will be made available by the authors, without undue reservation.
